# Determination of ATP Content in Cells

**DOI:** 10.1111/cpr.70184

**Published:** 2026-04-27

**Authors:** Boqiang Fu, Wenfeng Huang, Yingying Liu, Lei Wang, Yuan Liu, Jiani Cao, Wenjuan Duan, Aijin Ma, Hongling Zhao, Shuaishuai Niu, Shijun Hu, Qiyuan Li, Yong Zhang, Yaojin Peng, Xiaoyou Yu, Junying Yu, Jun Wei, Yu Zhang, Guoqiang Hua, Xin Liu, Changlin Wang, Tao Na, Yang Zhao, Jiaxi Zhou, Peng Xiang, Zhihong Wu, Qubo Chen, Peijun Zhai, Hengjun Gao, Jie Hao, Tongbiao Zhao, Jing Wang

**Affiliations:** ^1^ National Institute of Metrology Beijing China; ^2^ Standard Committee, Chinese Society for Cell Biology Shanghai China; ^3^ Institute of Zoology Chinese Academy of Sciences Beijing China; ^4^ Tsinghua University Beijing China; ^5^ Zephyrm Biotechnologies Co., Ltd. Beijing China; ^6^ National Institutes for Food and Drug Control Beijing China; ^7^ Promega (Beijing) Biotech Co., Ltd. Beijing China; ^8^ Beijing Technology and Business University Beijing China; ^9^ Beijing Institute for Stem Cell and Regenerative Medicine Beijing China; ^10^ Soochow University Suzhou China; ^11^ Shenzhen Medical Academy of Research and Translation Shenzhen China; ^12^ Shenzhen HHLIFE Co., Ltd. Shenzhen China; ^13^ Nuwacell Biotechnologies Co., Ltd. Hefei China; ^14^ D1 Medical Technology Shanghai China; ^15^ China National Institute of Standardization Beijing China; ^16^ Peking University Beijing China; ^17^ Institute of Radiation Medicine Chinese Academy of Medical Sciences Tianjin China; ^18^ Sun Yat‐sen University Guangzhou China; ^19^ Peking Union Medical College Hospital Chinese Academy of Medical Sciences Beijing China; ^20^ The Second Clinical College of Guangzhou University of Chinese Medicine Guangzhou China; ^21^ China National Accreditation Service for Conformity Assessment (CNAS) Beijing China; ^22^ Shanghai National Engineering Research Center of Biochip, Shanghai Engineering Center for Molecular Medicine Shanghai China; ^23^ University of Chinese Academy of Sciences Beijing China

## Abstract

The overlap HPLC chromatograms of ATP standard (red) and ATP in cells (blue). Poroshell 120 EC‐C18 (3 × 150 mm, 2.7 μm), 25°C, pH 6.8 buffer solution of 0.05 mol/L KH_2_PO_4_ ‐ 0.05 mol/L K_2_HPO_4_ (V:V = 1:1) as mobile phase, 0.6 mL/min, 254 nm.

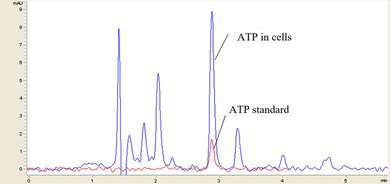

Measuring cell viability is essential for determining the physiological state of cells under experimental conditions and is widely and routinely used in basic research [1, 2], drug discovery [3, 4], cell banks and cellular therapeutic products [5, 6]. Cell viability can be assessed through a broad range of analytical methods that evaluate different attributes related to the viable state of the cell(s) [7]. ‘Determination of ATP content in cells’ is one of a series of analytical method standards developed for cell viability test. It is jointly drafted and agreed upon by the experts from the Standard Committee of the Chinese Society for Cell Biology. This standard specifies the terms and definitions, reagents, instruments, sample preparation, assay procedures, and data processing for determining ATP content in cells using High Performance Liquid Chromatography (HPLC) method and bioluminescence method, applicable to mammalian cells. It is originally released by the China Society for Cell Biology on 28 October 2024. This standard standardizes the determination of cell viability based on ATP concentration and will be beneficial for scientific research, quality control of cellular therapeutic products, and the control and release of cells in cell banks worldwide.

## 1 Scope

This document specifies the High Performance Liquid Chromatography (HPLC) method and bioluminescence method for determining ATP content in cells. It includes terms and definitions, reagents, instruments, sample preparation, assay procedures and data processing.

This document is applicable to the determination of ATP content in mammalian cells.

## 2 Normative References

There are no normative references in this document.

## 3 Terms and Definitions, Abbreviations

### 3.1 Terms and Definitions

#### 3.1.1 Adenosine 5′‐Triphosphate, ATP

A compound consisting of adenosine linked to three phosphate groups, which contains three high‐energy phosphate bonds that releases significant energy upon hydrolysis, and serves as the most direct source of energy in living organisms.

#### 3.1.2 ATP Bioluminescence

Luminescence produced by the reaction of adenosine 5′‐triphosphate (ATP) released from lysed cells with luciferin and luciferase.

### 3.2 Abbreviations

ATP: Adenosine 5′‐triphosphate

HPLC: High Performance Liquid Chromatography

PBS: Phosphate Buffer Solution

## 4 High Performance Liquid Chromatography Method

### 4.1 Principle

Cells are ultrasonically lysed with a perchloric acid aqueous solution to release ATP from living cells. After neutralization using a potassium carbonate aqueous solution, the ATP in the extract is determined by HPLC‐UV method [8, 9]. The amount of ATP in the cells is qualified by the external standard method with ATP purity reference material. Based on the total number of cells in the sample, the average amount of ATP per cell is calculated.

### 4.2 Reagents and Solutions

Unless otherwise specified, the reagents used in this method are analytical grade reagents, and the water is primary water as specified in GB/T 6682 Water for analytical laboratory use―Specification and test methods (ISO 3696: 1987, MOD).

1. Certified Reference Material of Adenosine 5′‐Triphosphate (ATP) Disodium Salt: the purity, calculated with adenosine 5′‐triphosphate disodium salt, shall be ≥ 92%, excluding crystalline water and adsorbed water.
2. Trypsin–EDTA.
3. Perchloric acid: Mass fraction 71%, molar concentration 12.44 mol/L.
4. Potassium dihydrogen phosphate: Purity of 99% or more.
5. Dipotassium hydrogen phosphate: Purity of 99% or more.
6. Potassium carbonate: Purity of 99% or more.
7. PBS buffer solution: pH = 7.4.
8. Mobile phase: Buffer solution of 0.05 mol/L KH_2_PO_4_ and 0.05 mol/L K_2_HPO_4_ (V:V = 1:1), pH = 6.8.
9. ATP standard stock solution (1.0 × 10^4^ μmol/L): Approximately 5.5 mg of ATP reference material is accurately weighed, dissolved in 1.0 mL water by vortex shaking. When stored at 4°C, it can be stably stored for 1 months without microbial contamination. It can be stored stably for 1 year at −20°C.
10. ATP series standard working solution: The ATP standard stock solution was diluted with the mobile phase (as described in Section 4.2: list no. 8) to prepare a series of ATP standard working solutions with concentrations of 100, 50, 10, 5 and 2 μmol/L.

### 4.3 Instruments

1. High‐performance liquid chromatograph: Equipped with a UV detector.
2. Vortex oscillator.
3. pH meter: Accuracy of 0.01.
4. Electronic balance: Accuracy of 0.1 mg.
5. Centrifuge: 4°C, centrifugal force ≥ 14,000 g.
6. Ultrasonic oscillator.
7. Pipette: Range of 10 to 100 μL and 100 to 1000 μL.
8. Cell counter.

### 4.4 Sample Preparation and Pre‐Treatment

#### 4.4.1 Collection of Cell Samples

##### 4.4.1.1 Suspension Cell

The cell suspension, containing approximately 2 × 10^7^ to 4 × 10^7^ cells, is centrifuged at 4°C and 460 g for 2 min. After centrifugation, the supernatant is discarded. Add 1 mL of pre‐cooled PBS to the cell pellet, resuspend the cells and centrifuge again under the same conditions. Discard the supernatant and repeat the washing step with another 1 mL of pre‐cooled PBS. Divide the cell suspension into three equal parts of 300 μL each, placing them into separate 1.5 mL centrifuge tubes. These samples can be frozen in liquid nitrogen for later ATP extraction.

Use the remaining approximately 100 μL of the cell suspension to measure cell concentration with a cell counter.

##### 4.4.1.2 Adherent Cell

The culture medium of adherent growth cells is removed and discarded, and then the cell culture flask is rinsed with pre‐cooled PBS, and 1 mL Trypsin–EDTA digestion solution is added. The cell culture flask is incubated in the cell incubator (37°C, 5% CO_2_) for 3 min. When the cell shape becomes round and the cell shedding occurs, 2 mL new culture medium is added immediately to stop digestion. Then the cells are treated according the procedure as described in Section 4.4.1.1.

#### 4.4.2 Extraction of Intracellular ATP

For cell precipitation obtained in Section 4.4.1, containing approximately (6 × 10^6^ ~ 1.2 × 10^7^) cells/tube, 500 μL perchloric acid aqueous solution (1 mol/L) is added to each cell sample. Ultrasonic extraction is carried out in ice bath for 2 min, and high‐speed vortex shaking for 2 min. Centrifuge at 13,500 g (4°C) for 5 min and remove the supernatant. 250 μL potassium carbonate aqueous solution (2 mol/L) is used to neutralize the solution. Centrifuge at 13,500 g (4°C) for 5 min. Take the supernatant and transfer to 3 centrifuge tubes. Rehydrate to about 1 mL separately (calculate the volume of the cell extract based on the solution mass and density). To do the filtration with 0.22 μm water filter membrane, the filtrate is measured immediately or stored below −70°C.

### 4.5 Sample Analysis

#### 4.5.1 Analytical Conditions of HPLC

1. HPLC column: Aqueous C18 column (3 mm × 150 mm, 2.7 μm).
2. Mobile phase: Buffer solution of 0.05 mol/L KH_2_PO_4−_0.05 mol/L K_2_HPO_4_ (V:V = 1:1), pH = 6.8.
3. Column temperature: 25°C.
4. Flow rate: 0.5 mL/min.
5. Detection wavelength: 254 nm.
6. Injection volume: 1 μL.

#### 4.5.2 ATP Standard Working Curve

The external standard method is used for quantification. ATP series standard working solution with the concentration of 2, 5, 10, 50, 100 μmol/L are injected into the HPLC column, and the peak areas of ATP are measured. A standard curve is drawn with the HPLC peak area as the ordinate and the concentration of the ATP series standard working solution as the abscissa, and a linear regression equation is fitted. The linear correlation coefficient *r* should be greater than 0.995.

#### 4.5.3 HPLC Analysis of ATP Content in Cell Sample Solution

Under the same HPLC conditions, the ATP extracts of cells obtained from Section 4.4.2 are analysed.

HPLC images of ATP standard solutions and ATP in cells are shown in Supporting Information: Appendix A.

### 4.6 Result Calculation

The ATP standard curve is drawn with the data measured in Section 4.5.2, and the linear regression equation is obtained. According to the HPLC peak area of ATP in the cell sample, the external standard method is used to calculate the ATP content of the measured cell population.

The total number of cells in each sample is calculated by measuring the cell concentration with a cell counter.

The average amount of ATP in individual cell of the sample is further obtained. The calculation formula is as follows:

$$n=\frac{c_{i}\times V_{1}/1000}{N}$$

where:

*n*—The average amount of ATP in a single cell in the sample, μmol.

*c*_*i*_—The concentration of ATP in the intracellular ATP extract, μmol/L.

*V*_1_—The volume of intracellular ATP extract, 1 mL.

*N*—The total number of cells in the sample.

### 4.7 Precision

The absolute difference between the results of two independent assays obtained under repeatable conditions must not exceed 20% of the arithmetic mean.

## 5 Bioluminescence Method

### 5.1 Principle

The cells are lysed using a cell lysis agent to release ATP in viable cells. The ATP extract is incubated with the assay kit solution containing luciferase and luciferin, and the intensity of luminescence light signal is measured by a tube type or microwell plate luminescence detector [3, 4]. ATP purity reference material is used as calibrator to qualify the total amount of ATP in population cells. According to the total number of cells in the sample, the average amount of ATP in a single cell of the sample cell population is calculated.

### 5.2 Reagents and Solutions

Unless otherwise stated, the reagents used in this method are analytical grade reagents, and the water is primary water as specified in GB/T 6682.

1. Certified reference material of adenosine 5′‐triphosphate (ATP) disodium salt.
2. Trypsin–EDTA.
3. PBS buffer solution (pH = 7.4).
4. EDTA aqueous solution (pH = 6.5).
5. Cell culture medium.
6. ATP bioluminescence test kit: Contains cell lysed agent and luminescent reagent, stored at −70°C.
7. ATP standard stock solution (1.0 × 10^4^ μmol/L): About 5.5 mg ATP standard material is accurately weighed and added to a centrifuge tube, 1.0 mL water is added with a pipette, and dissolved by vortex shaking. When stored at 4°C, it can be stably stored for 1 month without microbial contamination. It can be stored stably for 1 year at −20°C.
8. ATP series standard working solution: The ATP standard stock solution is diluted 10× with cell culture medium to obtain ATP series standard working solution with the concentration of 1000, 500, 200, 100, 50, 10 nmol/L.

### 5.3 Instruments

1. Tube type or microwell plate type luminescent detector.
2. Vortex oscillator or fixed orbit oscillator.
3. pH meter: Accuracy 0.01.
4. Electronic balance: Precision 0.1 mg.
5. Centrifuge: 4°C, centrifugal force ≥ 14,000 g.
6. Ultrasonic oscillator.
7. Cell incubator: 37°C, 5% CO_2_.
8. Pipette: 10–100 μL, 100–1000 μL.
9. Cell counter.
10. Opaque multi‐well plate: Compatible with microwell plate luminescence detector 96‐well plate/384‐well plate/1536‐well plate.

### 5.4 Sample Preparation

#### 5.4.1 Suspension Cell

For the suspended cells growing in cell culture flask, the cell suspension (approximately 1 × 10^4^–4 × 10^4^ cells) is transferred into a centrifuge tube and centrifuged at 460 g for 2 min at 4°C, and the supernatant is discarded. Then, 1 mL pre‐cooled PBS is added. The cells are resuspended and washed. The suspension is centrifuged at 460 g for 2 min at 4°C again, and the supernatant is discarded. Next, 400 μL of pre‐cooled PBS is added and the cells are resuspended again. The cell suspension is divided into three wells of a 96‐well plate at a volume of 100 μL/well (25 μL/well for 384‐well plate, 4 μL/well for 1536‐well plate). The samples are stored at −70°C until testing.

The remaining cell suspension, approximately 100 μL, is used to measure the cell concentration by a cell counter.

The cells grown in 96‐well plate, 384‐well plate and 1536‐well plate could be directly detected without treatment.

#### 5.4.2 Adherent Cell

For adherent cells growing in cell culture flask, the culture medium is removed and discarded, and the cell culture flask is rinsed with pre‐cooled PBS. Then, 1 mL Trypsin–EDTA digestion solution is added. To incubate in the cell incubator (37°C, 5% CO_2_) for 3 min. When the cell shape become round and the cell shedding occurred, 2 mL of fresh culture medium is added immediately to stop digestion. The cells are then treated according the procedure as described in Section 5.4.1.

Cells grown in 96‐well plate, 384‐well plate and 1536‐well plate could be directly detected without treatment.

### 5.5 Sample Analysis

#### 5.5.1 Bioluminescence Analysis of ATP Standard Solution

According to the protocol of ATP bioluminescence detection kit, ATP series standard working solution with the concentrations of 1000, 500, 200, 100, 50, and 10 nmol/L are prepared and added to the test tube or 96 well plate for 100 μL (25 μL for 384 well plate; 4 μL for 1536 well plate) with two parallel wells per concentration. An equal volume of ATP luminescence reagent is added to each well, and the test tubes or microwell plates are mixed on a vortex oscillator or a fixed‐orbit oscillator for 2 min, following 10 min incubation at room temperature to stabilize the luminescence signal. The luminescent intensity is measured using a tube luminescent detector or a microplate luminescent detector.

The standard curve is drawn with the luminescence intensity as the ordinate and the concentration of ATP series standard working solution as the abscissa, and the linear regression equation is fitted. The linear correlation coefficient *r* should be greater than 0.995.

#### 5.5.2 Bioluminescence Analysis of ATP Content in Cell Sample Solution

For the cell samples to be tested in Section 5.4, the same volume of ATP luminescence reagent is added to each test tube or sample well. The test tube or microwell plate is mixed in a vortex oscillator or a fixed orbital oscillator for 2 min to lyse the cells, and then incubated at room temperature for 10 min to stabilize the luminescence signal. The luminescent intensity is measured by tube luminescent detector or microplate luminescent detector. Three parallels are tested for each sample.

To prevent ATP contamination and degradation, experiments should be performed in a biosafety cabinet or super clean bench, wearing masks and gloves, and avoiding to contact with potentially contaminated surfaces and equipment. Gloves, laboratory surface and equipment are cleaned with a 10% bleaching solution and patted dry. Individually packaged or designated ATP‐free pipet and pipet aspirates should be used whenever possible.

### 5.6 Result Calculation

Using the linear regression equation of ATP standard curve in Section 5.5.1, the external standard method is used for quantification, according to the luminescence intensity of ATP in the sample, the total ATP content of the measured cell population is calculated.

The total number of cells in each sample is calculated by measuring the cell concentration with a cell counter.

The average amount of ATP in individual cell of the sample is further obtained. The calculation formula is as follows:

$$n=\frac{c_{i}\times V_{1}/1000000}{N}$$

where:

*n*—The average amount of ATP in a single cell of the sample, nmol.

*c*_*i*_—The concentration of ATP in intracellular ATP extract, nmol/L.

*V*_1_—The volume of intracellular ATP extract, μL.

*N*—The total number of cells in the sample.

### 5.7 Precision

The absolute difference between the results of two independent assays obtained under reproducible conditions must not exceed 20% of the arithmetic mean.

## Author Contributions

Boqiang Fu designed and established the method, and drafted the manuscript. Jing Wang, Tongbiao Zhao and Jie Hao contributed to conception and design. Wenfeng Huang, Yingying Liu, Yuan Liu, Wenjuan Duan contributed to method validation. Lei Wang, Jiani Cao, Aijin Ma, Hongling Zhao, and Shuaishuai Niu contributed to draft and revise the manuscript. Shijun Hu, Qiyuan Li, Yong Zhang, Yaojin Peng, Xiaoyou Yu, Junying Yu, Jun Wei, Yu Zhang, Guoqiang Hua, Xin Liu, Changlin Wang, Tao Na, Yang Zhao, Jiaxi Zhou, Peng Xiang, Zhihong Wu, Qubo Chen, Peijun Zhai and Hengjun Gao critically read and revised the manuscript.

## Funding

This work was supported by grants from the Basic Research Foundation AKYZD2407 from National Institute of Metrology China, the National Key R&D Program of China 2017YFF0204601.

## Supporting information


**Data S1:** Supporting Information.

## Data Availability

The data that support the findings of this study are available on request from the corresponding author. The data are not publicly available due to privacy or ethical restrictions.
