# Supplementary figures and images for: Determination of ATP Content in Cells
Source: Cell Prolif. 2026 Apr 27;59(7):e70184. doi: 10.1111/cpr.70184 (PMC13325470; doi:10.1111/cpr.70184)

**Annex A**

（Informative）

Analysis of ATP content in cells by HPLC


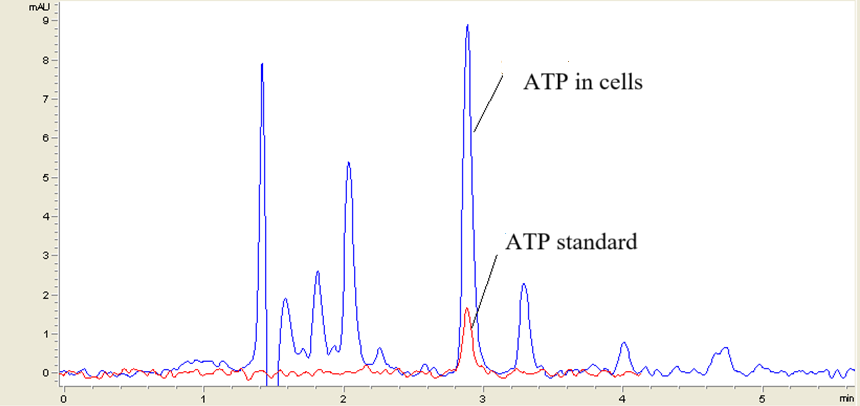

Supplement: Supplementary file 1 — Data S1: Supporting Information. [file CPR-59-e70184-s001.docx]
